# Sampling rate influences saccade detection in mobile eye tracking of a reading task

**DOI:** 10.16910/jemr.10.3.3

**Published:** 2017-06-07

**Authors:** Alexander Leube, Katharina Rifai, Katharina Rifai

**Affiliations:** Institute for Ophthalmic ResearchUniversity of Tuebingen, Germany

**Keywords:** eye movement, mobile eye tracking, saccades, reading

## Abstract

The purpose of this study was to compare saccade detection characteristics in two mobile eye trackers with different sampling rates in a natural task. Gaze data of 11 participants were recorded in one 60 Hz and one 120 Hz mobile eye tracker and compared directly to the saccades detected by a 1000 HZ stationary tracker while a reading task was performed. Saccades and fixations were detected using a velocity based algorithm and their properties analyzed. Results showed that there was no significant difference in the number of detected fixations but mean fixation durations differed between the 60 Hz mobile and the stationary eye tracker. The 120 Hz mobile eye tracker showed a significant increase in the detection rate of saccades and an improved estimation of the mean saccade duration, compared to the 60 Hz eye tracker. To conclude, for the detection and analysis of fast eye movements, such as saccades, it is better to use a 120 Hz mobile eye tracker.

## Introduction

The investigation of eye movements using eye tracking technology provides a powerful tool for different disciplines. Besides its role in scientific and clinical tasks, eye tracking applications are widely used for examining visual attention in marketing studies ( 1-3 ), adapting learning behavior in real time situations ( 4 ) or to enhance the control modalities in computergames ( 5-7 ). Especially saccadic eye movements and their statistics are of interest.

They are for instance used to investigate eye movements during reading, scene perception and visual search task, see Rayner ( 42 ) for review. Eye movement abnormalities like corrective saccades in a smooth pursuit task were shown to supplement clinical diagnosis of schizophrenia ( 8-10 ) and can be linked to cognitive deficits in word processing of schizophrenia patients ( 11 ). Furthermore, saccadic eye movements can be used as objective indicators in screening mental health ( 12 ) for instance of dyslexia ( 13-15 ) or autism ( 16, 17 ). This clearly shows the scientifically and clinically importance to detect the characteristics of saccades accurately. Such eye movement tests are often conducted on controlled conditions with high accuracy eye trackers that require head stabilization and presentation of stimuli on a fixed display. This, however, is unlike normal visual perception, and it is therefore important to move to more day-today tasks. Eye movements in such tasks can only be measured with mobile eye trackers, and it is unclear how well these can measure and detect saccadic eye movements.

In the analysis of eye tracking data, the algorithm used to detect events, such as fixations, blinks and saccades is the crucial factor. Algorithms can be classified in three main categories, based on their threshold criteria: dispersion, velocity or acceleration-based ( 18-20 ), or the combination of these criteria. The velocity-threshold identification is the fastest algorithm (no back-tracking required as in dispersion algorithms) that differentiate fixations and saccades by their point-by-point velocities and requires only one parameter to specify, the velocity threshold ( 18 ). When using velocity-based algorithms to analyze eye tracking data, the sampling rate of the eye tracking signal becomes the limiting factor ( 21 ). During saccades, the eye movements are very fast, and at low sampling rates, insufficient samples of these fast movements may be available for correct detection. Because of their velocity characteristics saccades can be classified as “outliers” in the velocity profile ( 22-24 ) and serve as a robust criteria in analyzing eye tracking data.

According to the Nyquist theorem, a higher sampled eye tracker detects saccades of shorter duration in comparison to a lower sampled eye tracker which saccade detection shows a minimum duration threshold of twice the Nyquist frequency. Thus, specifically an increase of sampling frequency from 60 Hz to 120 Hz is expected to increase the detection rate of saccades, whose durations are in the range between approximately 16 and 33 ms. The main sequence of saccades shows a linear relationship between saccade amplitude and duration ( 24, 25 ). In reading, which is a common activity and highly important in modern day-to-day life, saccade distributions show a high number of small saccades ( 33 ) which would not be detected if they fall into the interval between 16 and 33 ms. Saccadic behavior in reading tasks is a well-studied and explained characteristic of human eye movements . The task-specific saccade distributions directly impact the detection rate of eye trackers with a limited sampling rate. Thus, specifically in this task, an accurate choice of sampling frequency is crucial. Therefore, we assume that an eye tracker with higher sampling might detect more saccades in a reading task, as it will also detect short duration saccades. We furthermore hypothesize, that the estimation of mean saccade duration is more reliable when estimated from higher sampled gaze, because more samples will be available to reliably detect saccade start and end.Studies examining eye movements typically rely on high-sampling static eye trackers. But, novel mobile eye trackers allow recordings in more natural scenarios, especially paradigms in which the subject is freely behaving. With increasing use of mobile eye trackers, it becomes inevitable to evaluate how well mobile eye trackers can detect and measure saccadic eye movements. Thus, this study evaluates the impact of an increase in sampling rate of a head worn eye tracker designed for field studies from 60 Hz to 120 Hz in a realworld task with a topic of high research interest: reading ( 26 ).

## Methods

### Participants

11 eye-healthy participants with a mean age of 34.9 ± 9.9 years were included in the study. The participants had normal or corrected-to-normal vision. All participants were naïve to the purpose of the study. All procedures followed the tenets of the Declaration of Helsinki. Informed consent was obtained from all participants after explanation of the nature and possible consequences of the study.

### Equipment and experimental procedure

Participants were wearing one of two mobile eye trackers (SMI ETG w, 60 Hz sampling; SMI ETG 2w, 120 Hz sampling, SensoMotoric Instruments GmbH, Teltow, Germany). In order to evaluate saccade detection in these eye trackers, participants placed their head in a chin rest and a stationary eye tracker (EyeLink 1000, SR Research Ltd., Mississauga, Canada) was used as a reference. This eye tracker was placed below the screen at a distance of 60cm. For stimulus presentation, a visual display (VIEWPixx /3D, VPixx Technologies, Canada) at distance of 70 cm was used. Both mobile eye trackers and the stationary eye tracker were calibrated and validated using a 3-point calibration pattern composed of three black rings on a gray background. The stationary and mobile eye trackers recorded the eye positions simultaneously. The stationary eye tracker was set to record at a sampling rate of 1000 Hz, binocularly, and the two mobile eye trackers to either 60 Hz or 120 Hz binocular tracking. To minimize an influence of the IR signal from the stationary eye tracker on the tracking ability of the mobile eye tracking glasses, the power of the IR LED was reduced to a minimum of 50% intensity.

**Figure 1:**
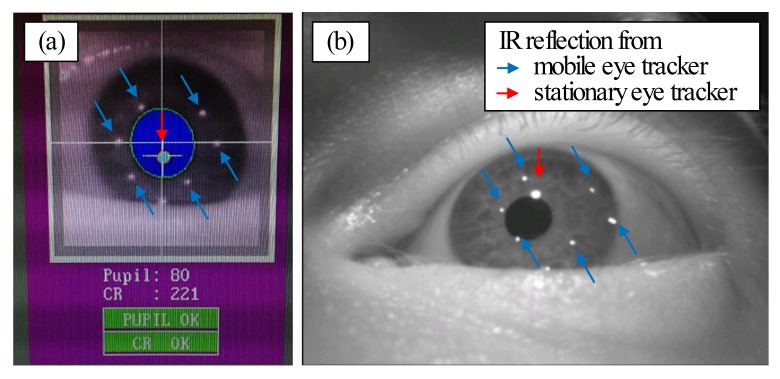
Comparison of the corneal infrared reflections from the stationary (red arrow) and the mobile (blue arrows) eye tracker. (a) represents the image from the stationary and (b) from the mobile eye tracker in simultaneous use.

The mobile eye tracking glasses use infrared (IR) LED´s arranged in a ring pattern within the glasses frame while the IR array from the stationary eye tracker is a single dot pattern. Because of the differences in shape and intensity of the reflection pattern (see Figure 1) and the intensity of the corneal reflex is much higher in the stationary eye tracker, both reflections were distinguishable from each other and a simultaneous measurement was possible. Moreover, both 60 Hz and 120 Hz eye trackers are expected to be equally affected by any potential mutal interference.

**Figure 2:**
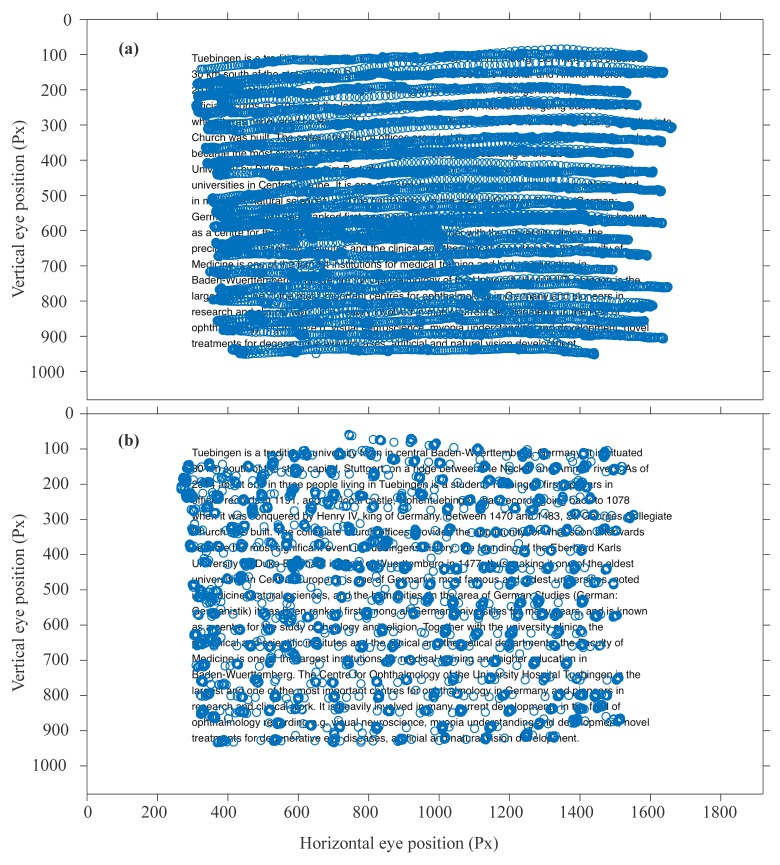
Raw data from gaze traces superimposed to the stimulus text. (a) illustrates the recorded eye position with a 1000 Hz eye tracker (EyeLink 1000) and in (b) at a sampling rate of 120 Hz (SMI mobile glasses). Gaze data and text were aligned manually by the author (horizontal and vertical stretching).

Prior to the experiment start, participants were informed that they will read a text, about which they will have to answer questions to ensure attentive reading. To enable an offline temporal synchronization between both eye trackers for the data analysis, a peripheral fixation point of 25° dislocation from the left side of the text was displayed for three seconds on the screen. Subsequently the sample text was presented. The letter size was set to 23 pixels, which corresponded to an angular size of 0.5 ° for capital letters using the font type Helvetica. A relatively large letter size ensured that every normally sighted participant was able to read the text. Two sample texts were created: Text 1 contained information about the human visual systems and covered 237 word, text 2 was about Tuebingen and the Eberhard Karls University Tuebingen with 276 words. An example of the experiment procedure is given in Figure 2. The participants were instructed to read silently and in their normal reading speed. Subsequently, the participants confirmed or rejected five statements regarding the content of the text, by pressing a button on the keyboard. All stimuli were programmed and displayed using the psychophysics toolbox (Psychtoolbox 3, Kleiner M, et al. 2007) in the Matlab programming language (Matlab, MathWorks Inc., Natick, Massachusetts).

### Analysis

From the eye tracking data the average number of fixations, their average duration and the average number of saccades and their durations were calculated. Blinks were excluded from the dataset prior to analysis. Blinks were identified on the basis of the individual eye tracker criteria (the pupil size is very small or either zero). Fixations and saccades were identified using an algorithm based on the velocity profile, see equation ( 1 ), of the gaze data calculated as the difference in horizontal eye position between successive positions and divided by the intersample time interval ( 18, 27 ) without application of a running-average filter prior to the analysis. According to equation (2), a fixation is classified as gaze points where the eye-velocity signal *v*^→^_*n*_ remains below a threshold of d = 60 °/sec for a minimum time duration of Δt_Fix_ = 100 ms (average fixation durations are around 200 – 300 ms ( 33 ; Starr & Rayner, 2001)). A single fixation and its associated duration was defined as the time interval where equation (2) resulted in a ‘false’ outcome. In addition to the fixation duration, the absolute number of fixations was analyzed.

**(1):**
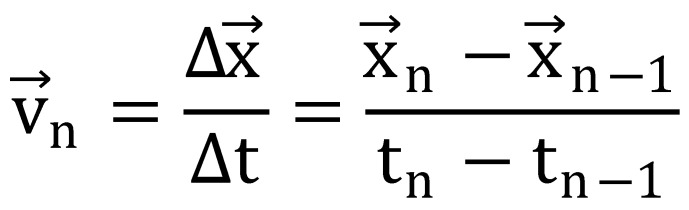


**(2):**
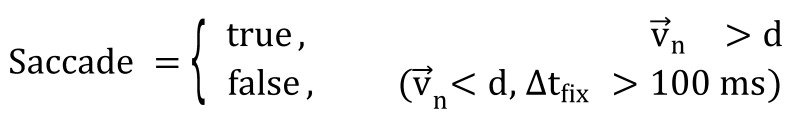


Furthermore, the velocity profile of the gaze data was used for saccade detection. A saccade event was identified as the time interval where the condition of equation (2) was true (i.e., the intervals not assigned to a fixation). The local maximum in this saccade time interval was localized using Matlab and marked a saccade event. The saccade duration was calculated as the time interval during which the velocity of the eye remained above the velocity threshold d and equation (2) was true.

To analyze the difference in performance between the 60 Hz and the 120 Hz mobile tracking glasses, the relative differences in the number and duration of detected fixations and saccades between the mobile and the stationary eye tracker were calculated and evaluated. All calculations consider the gaze data of the right eye. Normality of data was investigated using the Shapiro-Wilk test. In case of normal distributed data a t-test (power 1-β = 0.80) to test for difference in the detection ability was performed. Consequently, a Wilcoxon rank test in case of not normally distributed data was performed. The critical p-value (α error) was set to 0.05 and the statistical analyses was performed (IBM SPSS Statistics 22, IBM, Armonk, USA).

## Results

Figure 2 compares the eye movement data from the static eye tracker (a) and the data from the 120Hz mobile eye tracker (which is similar in the case of the 60 Hz mobile eye tracker) in (b), when superimposed on the text that was read. In order to plot the mobile eye tracking data (Figure 2b), the data were manually scaled using an empirically defined scaling factor. Figure 2 shows that the reduced sampling rate in the mobile eye tracker leads to a sparse representation of saccade midflight eye positions.

**Figure 3:**
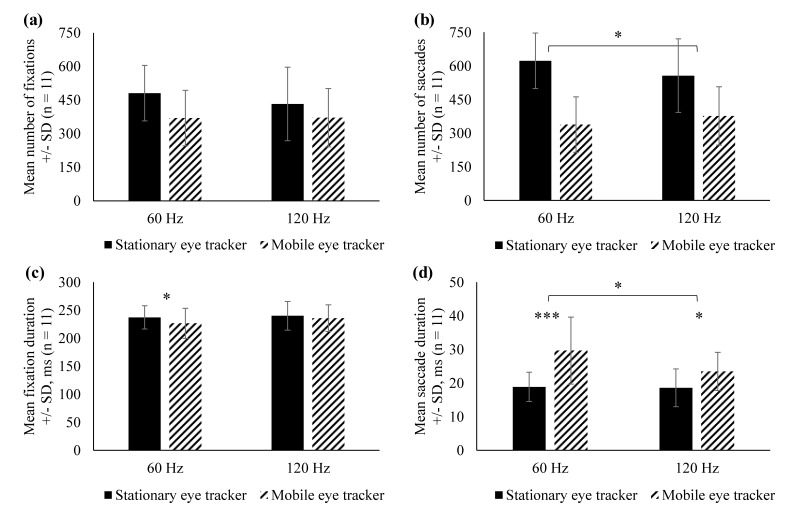
Mean number of fixations (a) and saccades (b), +/- standard deviation (SD). (c) and (d) present the mean fixation and saccade durations, respectively. Asterisks indicate the significance level: * α < 0.05, *** α < 0.001

A larger number of saccades were detected for the 120 Hz than for the 60 Hz eye tracker (p=0.011, two-sited t-test), see Table 1 and Figure 3. The 120 Hz mobile eye tracker also led to a more reliable estimation of mean saccade duration (Δ = 5.91 ms, p = 0.033, two-sited ttest), see Figure 3. Despite these differences, the number of saccades undetected by the stationary eye tracker but detected by the mobile eye trackers was very low and ranged below 1% of the total number of correctly detected saccades. The data therefore show that saccade detection was generally adequate in mobile eye trackers, the 120Hz eye tracker was better in measuring the duration of the saccade than the 60Hz eye tracker.

In contrast to the saccade, no significant difference in the number of fixations were found between the 60Hz and 120Hz eye tracker (p = 0.110, Wilcoxon-test). Statistical analysis showed no significant difference between the 60 Hz and the 120 Hz devices in fixation durations (p = 0.088, paired t-test). Nevertheless, there is a trend towards more accurate fixation detection in the 120 Hz device when compared to the stationary eye tracker. Mean and standard deviation of the number and the duration of saccades and fixations are shown in Figure 3.

**Table 1:** Relative comparison of two mobile eye trackers to a stationary eye tracker. Mean and standard deviation (SD) for the number and the duration of saccades and fixations. Asterisks indicate the significance level: * α < 0.05; n = 11

	60 Hz mobile eye tracker	120 Hz mobile eye tracker	Relative difference between mobile eye trackers
	Mean ± SD	
			
Number of saccades	56.11 ± 12.44 %	68.37 ± 13.97 %	12.25 % *
Duration of saccades (ms)	-10.81 ± 7.51 ms	-4.89 ± 2.76 ms	5.91 ms *
Number of fixations	76.72 ± 18.67 %	86.41 ± 15.43 %	9.69 %
Duration of fixations (ms)	10.55 ± 10.13 ms	4.30 ± 14.33 ms	6.25 ms

**Figure 4:**
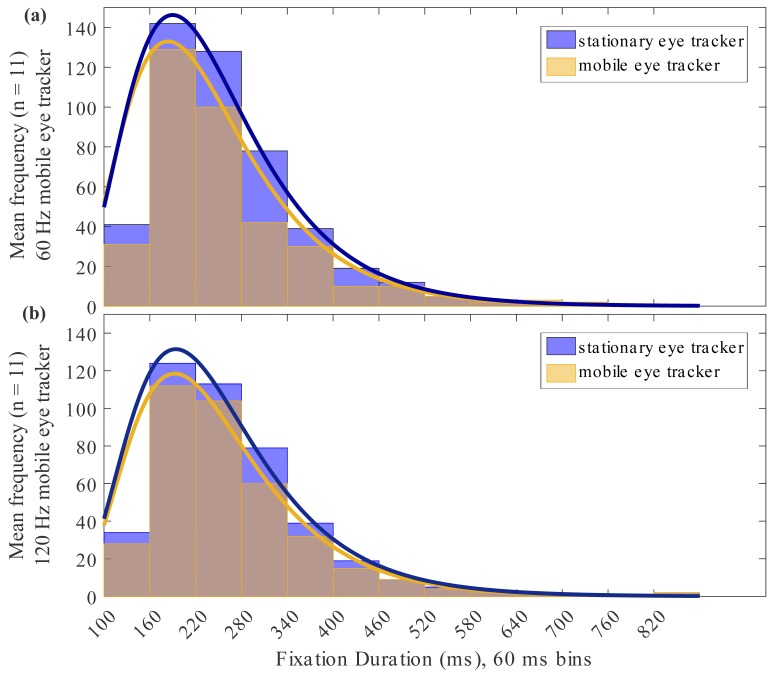
Mean frequency distribution (n = 11) of fixation durations for the stationary and the (a) 60 Hz and (b) 120 Hz mobile eye tracker in milliseconds (ms).

Figure 4 illustrate the frequency distribution of fixation durations in the 60 Hz (Figure 4a) and the 120 Hz devices (Figure 4b), respectively, compared to the stationary eye tracker. Fixation durations within the silent reading task ranged from 100 ms to 600 ms. The distribution of recorded fixations of the 60 Hz mobile eye tracker showed a shift of the maximum towards smaller fixation duration (p = 0.01, Wilcoxon rank test) while the distribution of the 120 Hz mobile eye tracker reveals a trend towards a better assessment of fixation durations (p = 0.59, Wilcoxon rank test), in comparison to the stationary reference eye tracker.

## Discussion

Previous studies have revealed that the use of stationary eye trackers with lower sampling rates results in significantly impoverished detection and measurement of saccadic eye movements, especially at the border of the stimuli screen ( 28 ). Generally, high-frequency stationary eye trackers should be preferred in investigations of saccades and the use of eye tracker with lower sampling rates should be restricted to the examination of fixation behavior and pupil size ( 29 ). While the effects of sampling rate for stationary eye trackers is known, no such information is available for the impact of mobile eye trackers, which place the cameras often closer to participants' eyes, use a different pattern of IR lighting, and a different calibration method. In the current study we compared the impact of sampling rate of mobile eye trackers on extraction rates of saccades and fixations in a reading task, as a common task of daily life.

## Mobile eye tracking and reading

The development of mobile eye trackers in the last years ( 30-32 ) has enabled researcher to examine eye movements during reading in a natural context ( 33 ). Mobile eye tracking of reading may enhance clinical diagnosis, for example, by differentiating progressive supranuclear palsy from Parkinson's disease ( 34 ) or for mental or linguistic disorders ( 11, 12 ). The measurement of eye movements in such tasks has led to the further understanding of learning ( 4 ) e.g. in medical and health professions ( 35 ) and can further be extended to e-learning applications ( 36 ). However, research on the reliability of mobile eye tracker in the detection of saccades and fixations especially in reading is sparse.

Current analysis of saccadic eye movements demonstrated the benefit of a higher sampling rate of the 120 Hz mobile eye tracker in the detection of saccades. During reading, the amplitude and number of both progressive and return (regression) saccades depend on various intrinsic and extrinsic factors ( 33 ). To evaluate these properties of saccades, it is therefore important to measure parameter of saccadic eye movements accurately. External aspects like visual information factors, e.g. the spaces or type of characters between words ( 37, 38 ) or the length and orthographic information of the words ( 39-41 ) impact saccadic amplitudes. Secondly, higher level factors, such as spatial coding ( 22 ) or the location of attention ( 42, 43 ) influence saccadic behavior. The main sequence saccadic eye movements ( 25, 44 ) demonstrates a linear correlation between saccadic amplitude and duration. In reading, people often make small saccadic eye movements (e.g. refixations of the same word), and therefore it is important to accurately detect small saccade amplitudes, it is crucial to use high-frequency equipment facilitate recording of small saccade durations. Our results revealed that saccades are better detected with a 120Hz sampling rate and that the distribution of saccade amplitudes is better measured with this higher sampling rate.

## Event detection algorithms for eye movement data

In the analysis of saccadic eye movements we used the standard approach based on the velocity profile of the gaze traces ( 18 ). Engbert and Kliegl ( 24 ) developed a velocity-based algorithm for the detection of microsaccades involving a noise dependent detection threshold and a temporal overlap criterion for the binocular occurrence of saccades. The advantage of using a noise dependent algorithm is that it can be adapted easily to the different eye tracking technologies and inter-individual differences ( 24 ). In future work, such noise dependent algorithms could therefore improve the detection performance of low sampled eye tracking data if the internal noise distribution is different between the eye trackers. A further approach for future work is to use data from both eyes in the analysis (the method by Engbert and Kliegl requires saccades to overlap in both eyes). However, in a real-world application a saccade detection algorithm could account for the binocularity and could increase accuracy of detecting saccades. One further extension is to use acceleration in addition to velocity to detect saccades ( 45 ) and combined this with noise-dependent saccade thresholds ( 46 ). Future work can also examine the use of more complex algorithms, including continuous wavelet and principal component analysis (PCA) or using that saccades can be identified as local singularities ( 47 ). Although the focus of the present study was not the comparison of algorithms for event detection in mobile eye tracking, advanced computations might improve the performance in event detection.

## Head-worn vs. head fixed eye tracking

The accuracy of eye tracker strongly depends on the conditions of the planned experiment ( 48 ) and furthermore on restrictions of head movements ( 49 ), hence it is important to consider application-oriented parameters as well. Generally, head-worn eye tracker are not restricted by a certain head position but the eye movements show a more complex pattern compared to a head-fixed situation, as for instance the vestibulo-ocular reflex ( 50 ) or the optokinetic nystagmus ( 51 ) occur. In inter-device comparisons between mobile eye trackers, further studies will have to clarify whether the event detection in mobile eye tracking depends on the type of tracking (e.g. pupil/glint tracking vs. 3d- eye model) or number of tracked eyes and the use of advanced calibration methods or algorithms, as discussed above. The current study reports on results of laboratory work including a head-fixed measurement setup. Mobile eye tracker enable a head-free acquisition of eye movement data and it was shown that head movements strongly contribute in the processing of the visual input, and thus to the oculomotor behavior ( 52, 53 ). Furthermore, in head-free scenarios position or orientation dislocation of the eye tracker on the head was shown to have significant influence on the accuracy of eye tracker ( 48 ). Given that, upcoming studies will have to investigate the sampling dependence of mobile eye tracker in head-free scenarios and real-world tasks.

## Fixation statistics in reading

Analysis of the fixation statistics during a common reading task showed no difference in the number of detected fixations or the mean fixation duration between the two mobile eye trackers. The observed mean fixation duration of 220 ms to 240 ms is comparable to other studies, which performed silent reading tasks ( 33, 54, 55 ). Yang et al. ( 38 ) reported shorter fixation duration for a reading task of 211 ms. The higher sampling rate of the 120 Hz mobile eye tracker led to a closer mapping of the frequency distribution of fixation durations, which is also represented in the significant smaller mean fixation duration, compared to the 60 Hz eye tracker. The frequency distribution of fixation durations showed in all cases the typical right tailed function ( 23, 38 ) with a maximum around 200 ms. The choice of a typical and realistic reading task, which represents a common daily visual duty, suggests that this estimation is also correct for the saccade amplitude distribution in reading and possibly in other tasks.

### Future implications in virtual reality applications

Mobile eye tracking is becoming progressively more important with the introduction of head-mounted-displays (HMD) and virtual reality (VR) glasses to enable a more realistic interaction mediated by human-computerinterfaces ( 56-61 ). Thus, there is an increasing need in eye trackers that combine usability for field studies with high accuracy and fast eye tracker in a miniaturized version and their incorporation into HMD or VR systems( 62, 63 ). Real time gaze estimation including precise and fast eye tracking enables accurate and thus comfortable stereo image presentation in virtual reality simulations or highly interactive virtual reality scenarios due to a higher sampling rate of eye tracker. Specifically, the detection of fast eye movements, like saccades, can enable gaze pointing to virtual objects. Juhola et al. ( 21 ) showed that a velocity based algorithm for saccade analysis requires a minimum of 70 Hz sampled data. DiScenna et al. ( 64 ) stated that for a reliable measurement of all kinds of eye movement video cameras with frame rates above 120 Hz are necessary. The results of the current study suggests that a 120 Hz mobile eye tracker leads to more reliable measurements also in a task specific evaluation of saccade and fixation statistics on reading.

## Conclusions

The study reports on a relative performance comparison between two mobile video-based eye trackers during reading. Low sampled eye tracking (60 Hz) lead to an under estimation of the detection of saccades while 120 Hz sampling results in a higher accuracy in the detection of fast eye movements and fixation durations. A certain detection of small saccade durations, as they occur in reading, requires higher sampling rates of the used eye trackers. Reliable and robust detection of saccades by fast and accurate mobile eye trackers will lead to novel developments in gaze-contingent protocols, e.g. for virtual reality simulations. Furthermore, increased sampling rates in eye tracking technology might enable advancements in new fields, such as in clinical applications for eye movement training scenarios in visual impaired patients or clinical eye movement marker analysis in diagnosis of diseases.

## Ethics and Conflict of Interest

The author(s) declare(s) that the contents of the article are in agreement with the ethics described in http://biblio.unibe.ch/portale/elibrary/BOP/jemr/ethics.html and that there is no conflict of interest regarding the publication of this paper.

## Acknowledgements

This work was done in an industry-on-campuscooperation between the University Tuebingen and Carl Zeiss Vision InternationalGmbH. The work was supported by third-party-funding (ZUK 63).The beta version of the 120 Hz mobile eye tracker was developed and friendly provided by the SensoMotoric Instruments GmbH, D14513 Teltow, Germany.
